# Validation of an online version of the rapid estimate of adult literacy in dentistry-30 for use by medical and dental students in Nigeria

**DOI:** 10.1186/s12903-024-04238-1

**Published:** 2024-04-22

**Authors:** Abayomi Abdul-Afeez Afolabi, Adetomiwa Oluwanifemi Afolabi, Morẹ́nikẹ́ Oluwátóyìn Foláyan

**Affiliations:** 1 Department of Community and Preventive Dentistry, Obafemi Awolowo University, Ile-Ife, Nigeria; 2 Faculty of Dentistry, Obafemi Awolowo University, Ile-Ife, Nigeria; 3 Department of Child Dental Health, Obafemi Awolowo University, Ile-Ife, Nigeria

**Keywords:** Oral health literacy, Self-reported oral health status, Medical and dental undergraduates

## Abstract

**Background:**

The need for online adaptations of research instruments became more pronounced amidst the COVID-19 pandemic. This study sought to modify the REALD-30 for online application (eREALD-30) and evaluate its content validity and internal reliability among medical and dental students in Nigeria.

**Methods:**

The eREALD-30 required participants to identify if each of the listed words were related to dentistry by ticking either a ‘yes’ or ‘no’ response with the option to mark ‘don’t know’ for words they were unsure about. Scores ranged from 0 to 30. Five oral health experts reviewed the eREALD-30 for cultural appropriateness, while content validity was evaluated by 10 medical and dental students. Internal reliability was assessed with 320 students recruited from 15 medical and dental schools spanning the country’s six geopolitical zones. These students also completed an oral health status assessment tool. Data collection utilized an online survey platform. Validity of the eREALD-30 was determined through correlation analysis between eREALD-30 scores and the oral health status assessment tool. Furthermore, binary logistic regression analysis was employed to explore the assocations between participants’ oral health status and their oral health literacy, adjusting for age, sex, and level of medical and dental education.

**Results:**

Out of the respondents, 178 (55.6%) exhibited poor oral health literacy, while 205 (64.1%) reported having good oral health status. Those with good oral health literacy demonstrated significantly higher odds of having good oral health status (OR: 1.61; 95% CI: 1.02–2.54; *p* = 0.04). However, individuals with good oral health literacy had increased odds of good oral health status after adjusting for confounding factors,, though this association did not retain statistical significance (AOR: 1.39; 95% CI: 0.86–2.24; *p* = 0.17). The eREALD-30 displayed strong internal consistency (Cronbach alpha = 0.933), indicating its reliability in assessing oral health literacy levels, alongside a high content validity score of 0.90.

**Conclusion:**

The study finding suggests that the e-version of the REALD-30 was reliable and valid for use among medical and dental students in Nigeria.

## Introduction

An individual’s oral health status is intricately tied to their level of oral health literacy [1]. This concept encapsulates the degree to which individuals possess the capacity to acquire, process, and understand essential health information and services needed to make informed decisions regarding oral health [2]. It involves the skill to comprehend written language, effectively communicate health-related information, navigate the healthcare system, and achieve and maintain good oral health [3]. Oral health literacy encompasses both the ability to access information and the proficiency to apply that knowledge in making informed decisions concerning oral health [4]. It serves as a strong predictor of an individual’s overall health, health-related behaviors, and health outcomes [5, 6].

Factors such as educational and socioeconomic status influence oral health literacy [7], which serves as a crucial factor in addressing oral health disparities and promoting the oral well-being of individuals [8]. Lower levels of oral health literacy have been associated with delayed diagnosis, insufficient adherence to medical guidelines, increased mortality risks, poorer health outcomes, and higher healthcare costs [9]. Additionally, individuals with inadequate oral health literacy are more likely to miss dental appointments [10], a recognized risk factor for heightened rates of dental caries [11] and compromised periodontal health [12]. Thus, it is imperative to identify individuals with low oral health literacy to develop programs aimed at reducing their susceptibility to oral diseases.

There are several tools for assessing oral health literacy, one of which is the Rapid Estimate of Adult Literacy in Dentistry (REALD)-30 [13]. This assessment tool aims to gauge dental word recognition and consists of 30 common dental terms with differing levels of complexity [13]. It is an interviewer-administered questionnaire that assesses reading comprehension, numeracy, listening, and decision-making skills [14]. The REALD-30 can be employed in studies involving adult populations or communities [15]. Participants are required to orally pronounce each word to the interviewer, with one point assigned for each correctly pronounced word. The overall oral health literacy score is calculated by summing the scores for all correctly pronounced words, resulting in a total score ranging from 0 to 30. The REALD-30 has demonstrated good reliability and validity [3, 16, 17].

However, with the emergence of the COVID-19 pandemic, face-to-face interactions became restricted, leading to a transition towards online platforms and self-administered questionnaires for epidemiological research [18]. Although phone interviews remained viable for questionnaire delivery, online surveys provided benefits such as wider outreach, cost-effectiveness, and reduced time requirements [19]. Therefore, an electronic rendition of the REALD-30 could improve the viability of conducting online investigations into oral health literacy.

To address this gap, the current study adapted the REALD-30 for online utilization, termed as the eREALD-30. Furthermore, there is a necessity to facilitate the collection of data on oral health status. Many surveys conducted in Nigeria have been sporadic and reliant on convenience sampling [20]. Most of the data originated from studies conducted in the southwest region of Nigeria or focused on specific sub-populations such as pregnant women and school-aged children [20, 21]. There is a scarcity of studies on oral health literacy in Nigeria. The limited research that exists utilized the REALD-30 without validation evidence for any population in Nigeria, typically conducted in hospital settings, often confined to a single hospital [22–24]. However, these studies indicated that low oral health literacy correlated with fair/poor oral hygiene status, gingivitis [4], suboptimal periodontal health [22], limited periodontal health knowledge [22], inadequate oral self-care behavior [22], substandard dental service utilization [22], and dental anxiety [23]. A validated tool for assessing oral health literacy is imperative to support epidemiological investigations in Nigeria. Thus, the aim of this study was to ascertain the internal reliability and content validity of the eREALD-30 as an appropriate instrument for evaluating oral health literacy among undergraduate medical and dental students in Nigeria.

## Methods

### Ethical consideration

The study obtained approval from the Health Research Ethics Committee of the Institute of Public Health at Obafemi Awolowo University (IPH/OAU/12/2024), ensuring adherence to ethical guidelines. All participants provided informed consent, which included detailed explanations of the study’s objectives, potential risks and benefits, voluntary participation, and the right to withdraw at any time. To safeguard participant confidentiality, personal identifiers such as names were not collected. Participants did not receive any direct benefits or compensation for their participation in the study.

### Study Design

This study was conducted as a cross-sectional analysis. The content validity and internal reliability of the eREALD-30 were assessed among medical and dental students in Nigeria.

### Survey instruments

The survey comprised three sections. The initial part of the questionnaire gathered data concerning participants’ socio-demographic profiles. The second section contained the eREALD-30 assessment. Lastly, the third section included the oral health status evaluation tool. Please refer to Appendix 1 for further details.

#### Socio-demographic information

The sociodemographic information sought were details on the participant’s age, sex, name of institution and current educational level in the medical and dental school.

#### Online Version of the Rapid Estimate of Adult Literacy in Dentistry (eREALD-30)

The eREALD-30 comprises 30 dental health-related terms arranged according to their level of difficulty. In the eREALD-30, these terms were presented in the online questionnaire, and participants were instructed to indicate whether each listed word pertained to dentistry by selecting either a ‘yes’ or ‘no’ response. Additionally, participants had the option to skip words by selecting ‘don’t know’. Each ‘yes’ response was scored as ‘1’ for the corresponding word, while ‘no’ and ‘don’t know’ responses were scored as ‘0’. The potential score range was from 0 to 30, with higher scores indicating higher levels of oral health literacy. To categorize respondents’ oral health literacy levels into good and poor, the mean score (15) was utilized. Scores equal to or below the mean indicated poor oral health literacy, while scores above the mean indicated good oral health literacy.

#### Oral Health Status Assessment

Data regarding participants’ oral health status was gathered to assess the validity of the eREALD-30 by correlating eREALD-30 scores with respondents’ self-reported oral health status [24]. Oral health was evaluated using a self-report questionnaire for dental health status assessment tool [25], comprising 10 items such as “How often do you brush your teeth during the day?“, “Do you use toothpaste containing fluoride?“, “Do you visit a dentist regularly for check-ups?“, “Do you experience bleeding while brushing your teeth?“, among others. Each affirmative response was scored as ‘1’, reflecting good oral health behavior/status, while poor oral health behavior/status received a score of ‘0’. The score ranged from 0 to 10, with higher scores indicating better oral health status. To categorize respondents’ oral health status into good and poor, the mean score (5) was utilized. Scores equal to or below the mean indicated poor oral health status, while scores above the mean indicated good oral health status. Tts sensitivity score is 85.1% and false positive rate is 29% [25]. The tool had however, not been validated for use in Nigeria.

### Validation of Survey Instrument

*Step 1: Development of the eREALD-30* – The content of the REALD-30 was retained. The eREALD-30 was then formatted into an online version. However, for the eREALD-30, respondents were required to write down a word that spontaneously comes to mind for each of the 30 words.

*Step 2: Expert Review of eREALD-30* - Five experts specializing in cariology, periodontology, community dentistry, and oral medicine were tasked with evaluating the online version of the eREALD-30. These experts were selected for their qualifications as practicing dentists within academia, boasting a publication history of at least 25 works in their respective fields and a teaching tenure of no less than 10 years in Nigeria. Their considerable expertise had been cultivated through extensive practical experience and formal education, making them well-equipped to accurately convey terminology within the Nigerian context. Their feedback was instrumental in refining the content’s structure to ensure a gradual progression in terminological complexity. Moreover, they meticulously reviewed spellings, wording, and language to ensure cultural relevance in the questionnaire. No unique comments were received.

*Step 3: Content validity* – On December 28th, 2022, the online version of the questionnaire was distributed to a group of ten medical and dental students, purposefully selected to participate in the survey and complete the form. Attention was given to ensuring representation from all five tiers of medical and dental education, as well as maintaining gender balance among participants. Each student was instructed to evaluate the relevance of every word in the survey using a 4-point scale ranging from least (scored 1) to most (scored 4) relevant. Subsequently, the content validity index for the eREALD-30 was calculated. Four students provided unique comments, which led to some adjustments in the questionnaire sequence, particularly in the placement order of the eREALD-30, and the inclusion of clearer instructions for respondents on how to complete the eREALD-30. The content validity index was computed, resulting in an overall value of 0.78, which was considered satisfactory based on established criteria [26].

*Step 4: Internal reliability* - Participants for the survey were recruited from 15 medical and dental schools spanning the country’s six geopolitical zones. Support from contacts within each school facilitated the recruitment process, ensuring representation across various academic levels ranging from 200 to 600 levels. These contacts received periodic reminders to continue recruiting respondents as necessary. Eligibility criteria for the reliability assessment included individuals aged 18 years and above, residing in Nigeria, and providing informed consent for study participation. Specifically, only medical and dental students currently enrolled in Nigerian medical and dental schools were considered for inclusion in the study, with no exclusion criteria in place. There were 320 participants engaged with assessing the internal reliability of the eREALD-30.

Data collection was conducted using the online survey platform Survey Monkey®. Survey links were configured to maintain respondent anonymity, permitting participants to freely modify their answers before submission, and imposing no time constraints. Each electronic device was restricted to submitting the survey only once. Administered in English, the questionnaire links were distributed to eligible participants—those aged 18 years and above, capable of giving consent, and proficient in reading the survey—via emails and social media platforms accessible to medical and dental students. The survey remained open for participation from January 24th, 2023, to May 19th, 2023.

### Data analysis

We assessed the internal reliability of the eREALD-30 by computing Cronbach’s alpha, a metric indicating internal consistency. The classification method for Cronbach’s alpha was based on the modified Landis and Koch categorization, as utilized by El Tantawi et al. [27]. Specifically, Cronbach’s alpha values falling between 0 and 0.39 were categorized as indicating a low level of internal consistency, those within the range of 0.40 to 0.79 were considered to demonstrate a moderate level, and values ranging from 0.81 to 1 were deemed to signify an excellent level of internal consistency. To ascertain the validity of the eREALD-30, we established correlations between the eREALD-30 scores and the scores obtained from the oral health status assessment.

The collected data underwent meticulous evaluation for completeness, with only fully completed datasets considered for entry and subsequent analysis. The analysis was conducted utilizing IBM SPSS (version 26.0). Descriptive statistics, including frequency, counts, and percentages, were utilized to depict the socio-demographic characteristics of the participants, as well as to outline the distribution of the primary study variables. Furthermore, a binary logistic regression analysis was carried out to examine the relationship between participants’ oral health status and their oral health literacy. The regression model was adjusted for age, sex, and level of medical and dental education.

## Results

Table 1 highlights the Content Validity Index for each component of the eREALD-30. The Item Content Validity index (I-CVI) of individual items reflects a substantial level of validity, except for items 9 (Sealants), 20 (incipient), and 29 (Apicectomy), which exhibit lower validity. The Scale Content Validity Index Average (S-CVI/Ave) aggregates to 0.90, signifying a strong overall validity for the instrument.

**Table 1:** Content validity index of eRapid estimate of adult literacy in dentistry-30 (*N* = 10)

Variable	I-CVI
Sugar	1.00
Smoking	0.90
Flossing	1.00
Brush	1.00
Braces	0.90
Pulp	0.90
Denture	1.00
Enamel	0.90
Sealant	0.60
Genetics	0.80
Caries	1.00
Restoration	0.90
Fluoride	1.00
Plaque	0.90
Extraction	1.00
Periodontal	0.90
Fistula	0.80
Cellulitis	0.80
Abscess	1.00
Incipient	0.60
Halitosis	1.00
Malocclusion	0.90
Gingiva	1.00
Dentition	1.00
Bruxism	1.00
Hyperemia	0.90
Analgesia	0.90
Hypoplasia	1.00
Apicectomy	0.50
Temporomandibular	0.80

Table 2 displays the socio-demographic attributes of the 320 participants involved in evaluating the instrument’s reliability. Among them, 255 (79.7%) were aged 17–24 years, 167 (52.2%) were female, 182 (56.9%) were pursuing medicine, 170 (53.1%) hailed from the southwest geopolitical zone, and 90 (28.1%) were in their second year of study.

**Table 2:** Respondents’ socio-demographic characteristics of medical and dental students in Nigeria (*N* = 320)

VARIABLES	FREQUENCY *N* = 320	PERCENTAGE (%)
Geopolitical Zone		
North Central Nigeria North East Nigeria North West Nigeria South East Nigeria South South Nigeria South West Nigeria	81 19 25 06 19 170	25.3 5.9 7.8 1.9 5.9 53.1
Course of study		
Medicine Dentistry	182 138	56.9 43.1
Year of Study		
Second Year Third Year Fourth Year Fifth Year Sixth Year	90 65 51 40 74	28.1 20.3 15.9 12.5 23.1
Age		
17–24 years 25–29 years 30–34 years 35–40 years	255 62 02 01	79.7 19.4 0.6 0.3
Sex		
Male Female	153 167	47.8 52.2

**Fig. 1:**
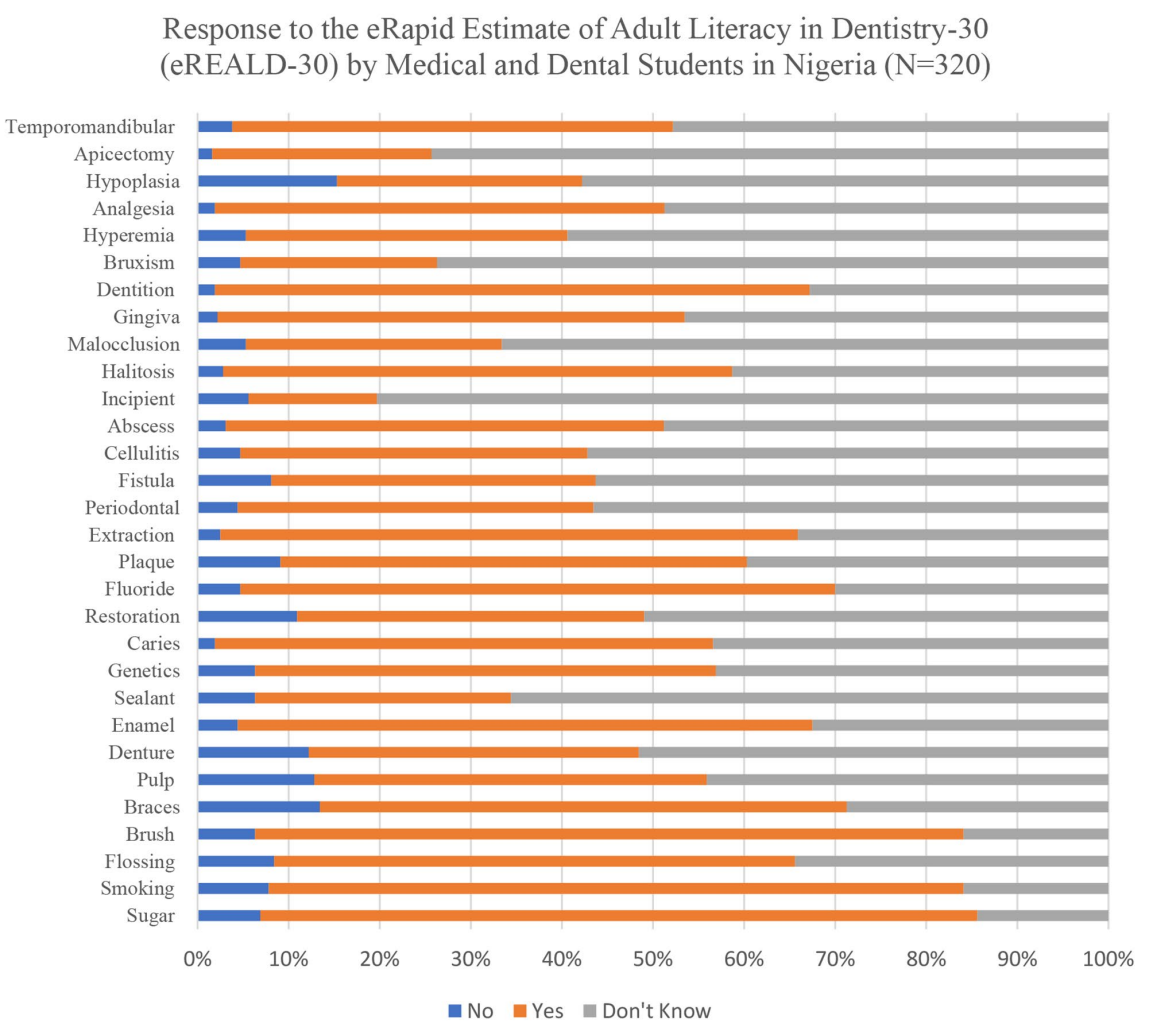
Response to the eRapid estimate of adult literacy in dentistry-30 (eREALD-30) by medical and dental students in Nigeria (*N* = 320)

Figure 1 presents a summary of responses to the eREALD-30. Specifically, 252 (78.8%), 244 (76.3%), and 249 (77.8%) of respondents identified the terms sugar, smoking, and brush as dental terminologies, respectively. Moreover, 202 (63.1%), 209 (65.3%), 203 (63.4%), and 209 (65.3%) also recognized enamel, fluoride, extraction, and dentition as dental terminologies, respectively. Conversely, terms such as bruxism, apicectomy, and incipient were not associated with dentistry.

Table 3 illustrates that 178 (55.6%) respondents exhibited poor oral health literacy, while 205 (64.1%) respondents reported good oral health status. Individuals with good oral health literacy displayed statistically significant higher odds of good oral health status (OR: 1.61; 95% CI: 1.02–2.54; *p* = 0.04).

**Table 3:** Association between the Level of Oral Health Literacy and Oral Health Status among Medical and Dental Students in Nigeria (*N* = 320)

Level of Oral health literacy	Level of Oral Health status		Total	OR (95% CI; *p* value)	AOR (95% CI; *p* value)
	Poor	Good			
Poor Good	70 (39.3%) 45 (31.7%)	108 (60.7%) 97 (68.3%)	178 (55.6%) 142 (44.4%)	1 1.61 (1.02–2.54; 0.04)	1 1.39 (0.86–2.24; 0.17)
Total	115 (35.9%)	205 (64.1%)	320 (100.0%)		

Upon adjusting for confounding factors, individuals with good oral health literacy still demonstrated increased odds of good oral health status, although the association no longer reached statistical significance (AOR: 1.39; 95% CI: 0.86–2.24; *p* = 0.17).

Table 4 displays the outcomes of the internal reliability assessment for the two instruments utilized in this study. The Cronbach’s alpha score for the eREALD-30 was 0.9333, signifying a high level of internal consistency and excellent reliability for the instrument. In contrast, the Cronbach’s alpha score for the oral health status assessment tool was 0.194, indicating poor reliability of the instrument due to its low value.

**Table 4:** Internal reliability analysis for the eREALD-30 and the oral health status assessment tools (*N* = 320)

Variables	Cronbach Alpha	Number of Items	Remark
Oral health literacy (eREALD-30)	0.933	30	Excellent level
Oral Health Status Assessment	0.194	10	Low level

## Discussion

In this study, we validated an online adaptation of the REALD-30 questionnaire, with findings indicating high internal consistency, thus affirming the reliability of the eREALD-30 [28]. Moreover, the tool exhibited substantial content validity, although items 9, 20 and 29 yielded lower CVI scores. In addition, the lack of a significant correlation between oral health status score and eREALD-30 score suggests a potential limitation in the tool’s capacity to distinguish between individuals with differing oral health status. It’s crucial to interpret this constrained discriminatory ability of the eREALD-30 cautiously, particularly considering the low reliability score observed in the oral health status assessment tool.

One of the strengths of the current study was the adaptation of a traditionally face-to-face tool for online utilization, which holds particular significance during events such as the COVID-19 pandemic, where face-to-face interactions are restricted. The successful validation of the online version of REALD-30 provides a critical resource for conducting remote studies, ensuring the continuity of data collection even amidst challenging circumstances. Additionally, we employed a tailored oral health assessment tool to ascertain the oral health status of study participants. Moreover, we recruited a participant population familiar with medical terminology, facilitating a clear comprehension and application of oral health concepts and its relevance [29]. Moreover, this tool could serve as a cost-efficient screening instrument to identify individuals requiring oral health care, especially in remote and hard-to-reach communities, particularly in resource-constrained settings, as low oral health literacy is associated with missed dental appointments [30]. Consequently, the tool could potentially furnish valuable insights to oral health authorities for crafting more tailored educational strategies for oral health within these communities. Nevertheless, a notable limitation may arise from the restricted access to internet facilities in such remote areas and among rural populations.

However, the study encountered a few limitations. Firstly, it was confined to medical and dental students, thereby constraining its generalizability to other populations. Medical and dental students were chosen for validating the inaugural online version of the REALD-30 due to their professional expertise, educational background, accessibility, and the practicability of conducting the study with this specific cohort. Their input could significantly enhance the refinement and validation of the test for both clinical practice and research purposes. Moreover, the study solely involved participants from Nigeria, restricting its applicability beyond countries with socioeconomic profiles akin to Nigeria’s. Additionally, the sample was obtained via convenience sampling, potentially biasing towards participants with higher socioeconomic status who possess smartphones and internet access. Consequently, this further impedes the generalizability of the findings to all medical and dental students hailing from households with lower socioeconomic status. Despite these limitations, the study yields novel insights.

First, the robust internal reliability and content validity of the eREALD-30 indicate that the items within the instrument effectively measure various dimensions of the same trait or construct, which is deemed acceptable [31]. These metrics also affirm the reliability and quality of the eREALD-30 for implementation among medical and dental students in Nigeria. Nonetheless, establishing the validity of the eREALD-30 across diverse cultures, age brackets, and educational backgrounds remains imperative. Achieving this necessitates meticulous translation and adaptation of the instrument, along with pre-testing and cognitive interviews conducted on the target populations, albeit this process poses its own set of challenges [32].

Second, our analysis revealed a low reliability of the oral health status assessment tool. This could be attributed to various factors such as a limited number of questions, weak interconnections among items, or the presence of diverse constructs within the tool [32]. We tried to mitigate the risk of using a tool with low reliability for this study by using an oral health status assessment tool that had been validated, since reliability is part of the assessment of validity [33]. This study finding underscores the possible need for developing a reliable oral health status assessment tool for medical and dental students in Nigeria. Self-reported oral health status may be a viable alternative method. Previous research has indicated that self-reported assessments of oral health status can be dependable and accurately reflect clinical conditions [34–37]. Utilizing self-reported measures of oral health offers a more convenient and cost-effective approach to evaluating oral health conditions across diverse populations and demographics, necessitating fewer resources and shorter research durations [37]. Notably, self-reported oral health status has already been utilized to assess the oral health of the general population in Nigeria [38, 39]. Additionally, single-item assessments of subjective health and well-being have exhibited enhanced validity in predicting health-related behaviors [40].

Third, the absence of a statistically significant association between poor oral health literacy and poorer oral health status, even after adjusting for confounders, sharply contrasts with the prevailing body of literature, which consistently demonstrates a strong link between oral health literacy and self-reported oral health conditions [4, 41–43]. This discrepancy in findings may stem from variations in methodologies, assessment tools, and the demographic composition of the study populations. Previous investigations typically encompass general populations or patients attending dental clinics, whereas our study specifically targeted medical and dental students. It’s plausible that the oral health literacy levels of our participants were already relatively high owing to their status as undergraduate medical and dental students. These students undergo comprehensive education and training in oral health as an integral part of their curriculum, which could have positively influenced their oral health literacy levels without necessarily leading to improved oral health status.

Fourth, nearly half of the participants displayed inadequate levels of oral health literacy. Considering the educational attainment of undergraduate students, one might expect that the concept and importance of oral health would be readily comprehensible and applied, regardless of their field of study [44]. However, our findings challenge this assumption. Prior studies have already suggested that a significant proportion of undergraduates have never sought dental care [45, 46]. The medical and dental schools targeted in our survey were those housing both medical and dental institutions, potentially granting medical students enhanced access to dental education and services. Further investigation is warranted to elucidate the factors contributing to the heightened rates of poor oral health literacy among medical and dental students in Nigeria.

The findings of this research hold several important implications. Firstly, the successful validation of the online adaptation of the REALD-30 questionnaire underscores its reliability, particularly evident in its outstanding internal consistency. However, the lower scores for items 9, 20 and 29 suggests potential limitations in the tool’s capacity to accurately gauge various levels of oral health literacy. Specifically, procedures like fissure sealants and apicectomy are not commonly provided by dentists in Nigeria and so may not be part of the routine oral health vocabulary of the populace. Incipient caries is also not a routine terminology; the synonym ‘enamel caries’ is more often used. The high ‘don’t’ know’ response points to this. Consequently, if these items yield low scores among medical and dental students, there is a risk that such scores may disproportionately influence the results of the eREALD-30 for the broader population, inaccurately reflecting levels of oral health literacy. Thus, it might be prudent to develop a culturally relevant oral health literacy tool with content tailored to specific contexts, as indicated by these findings of the content validity index. A validation process involving non-dental experts may help with adapting the eREALD-30 to be culturally appropriate wherever it would be used.

## Conclusions

In conclusion, this study highlights the internal reliability and structural content validity, along with the moderate to high individual content validity, of the adapted REALD-30 for online use (eREALD-30) when administered to medical and dental students in Nigeria. Nonetheless, it is imperative to validate the tool with diverse population groups to ensure its applicability and relevance for future online research, especially in scenarios such as pandemics where conventional face-to-face data collection may pose challenges. Additionally, future studies should delve into examining the correlation between oral health literacy and oral health status to uncover potential indicators of poor oral health status in Nigeria.

## Data Availability

All the data related to this study are presented in the results.

## Abbreviations

AOR: Adjusted Odds Ratio
CI: Confidence Interval
COVID-19: Corona Virus Infectious Disease 2019
eREALD-30: Online version of Rapid Estimate of Adult Literacy in Dentistry
REALD-30: Rapid Estimate of Adult Literacy in Dentistry
OR: Odds Ratio
