# Correction: Validation of an online version of the rapid estimate of adult literacy in dentistry-30 for use by medical and dental students in Nigeria

**DOI:** 10.1186/s12903-024-04488-z

**Published:** 2024-06-24

**Authors:** Abayomi Abdul-Afeez Afolabi, Adetomiwa Oluwanifemi Adedire, Morẹ́nikẹ́ Oluwátóyìn Foláyan

**Affiliations:** 1 Department of Community and Preventive Dentistry, Obafemi Awolowo University, Ile-Ife, Nigeria; 2 Faculty of Dentistry, Obafemi Awolowo University, Ile-Ife, Nigeria; 3 Department of Child Dental Health, Obafemi Awolowo University, Ile-Ife, Nigeria

**Correction to: BMC Oral Health (2024) 24:485**

10.1186/s12903-024-04238-1

In this article [1], the author name Adetomiwa Oluwanifemi Adedire was incorrectly written as Adetomiwa Oluwanifemi Afolabi.
